# Investigating preparation and characterisation of diphtheria toxoid‐loaded on sodium alginate nanoparticles

**DOI:** 10.1049/nbt2.12088

**Published:** 2022-05-24

**Authors:** Samira Aghamiri, Mojtaba Noofeli, Parvaneh Saffarian, Zahra Salehi Najafabadi, Hamid Reza Goudarzi

**Affiliations:** ^1^ Department of Biology Science and Research Branch Islamic Azad University Tehran Iran; ^2^ Department of Research and Development Razi Vaccine and Serum Research Institute Agriculture Research Education and Extension Organization (AREEO) Karaj Iran; ^3^ Department of Human Bacterial Vaccine Razi Vaccine and Serum Research Institute Agriculture Research Education and Extension Organization (AREEO) Karaj Iran

**Keywords:** antigen delivery system, diphtheria toxoid, loading capacity, loading efficiency, nanoparticles, sodium alginate

## Abstract

This paper aims to investigate the preparation and characterisation of the alginate nanoparticles (NPs) as antigen delivery system loaded by diphtheria toxoid (DT). For this purpose, both the loading capacity (LC) and Loading efficiency (LE) of the alginate NPs burdened by DT are evaluated. Moreover, the effects of different concentrations of sodium alginate and calcium chloride on the NPs physicochemical characteristics are surveyed in addition to other physical conditions such as homogenization time and rate. To do so, the NPs are characterised using particle size and distribution, zeta potential, scanning electron microscopy, encapsulation efficiency, in vitro release study and FT‐IR spectroscopy. Subsequently, the effects of homogenization time and rate on the NPs are assessed. At the meantime, the NPs LC and efficiency in several DT concentrations are estimated. The average size of the NPs was 400.7 and 276.6 nm for unloaded and DT loaded, respectively. According to the obtained results, the zeta potential of the blank and DT loaded NPs are estimated as −23.7 mV and −21.2 mV, respectively. Whereas, the LC and LE were >80% and >90%, in that order. Furthermore, 95% of the releasing DT loaded NPs occurs at 140 h in the sustained mode without any bursting release. It can be concluded that the features of NPs such as morphology and particle size are strongly depended on the calcium chloride, sodium alginate concentrations and physicochemical conditions in the NPs formation process. In addition, appropriate concentrations of the sodium alginate and calcium ions would lead to obtaining the desirable NPs formation associated with the advantageous LE, LC (over 80%) and sustained in vitro release profile. Ultimately, the proposed NPs can be employed in vaccine formulation for the targeted delivery, controlled and slow antigen release associated with the improved antigen stability.

## 1 INTRODUCTION

Over the last decades, using of nanoparticles (NPs) such as polymeric and liposomal NPs have been increased as new adjuvants and drug or antigen delivery systems [1, 2]. The most commonly used are the aluminium‐based adjuvants apart from directing immune system, have some disadvantages [3]. They are not equally efficient for all antigens due to their restricted immune‐enhancement effects and local reactions such as redness, swelling and pain at the injection site, or systemic reactions such as fever, chills and body aches associated with unfavourable immunoglobulin E responses [4, 5, 6]. Considering the above‐mentioned weak points for alum‐based adjuvants, biodegradable NPs as an adjuvant were developed by numerous procedures [7, 8, 9, 10, 11]. One of the important challenges in therapeutics is to deliver drug to the target site and preserve the necessary dosage in a sufficient period to achieve the desired medical outcome [12]. Nanoparticles used as the delivery systems have shown a protective impact on drug or antigen from degradation in physiological conditions, control release of drug, improve stability, antigen delivery to the antigen‐presenting cells (APCs), controlled‐release properties for the encapsulated antigens and as adjuvant role to improve the immune responses including the immunoglobulin type and subtypes depending on the helper T lymphocyte activation [2, 13, 14, 15, 16, 17].

It is worth noting that NPs used for antigen or drug delivery usually consist of three different aspects as (1) constituent composition of NPs such as natural polymers, synthetic polymers, lipids, etc.; (2) immunogenic or immune‐modulatory agents such as antigens, siRNA, etc. (3) immune‐stimulatory ligands attached to the particle surface like immune specific ligands, pathogen associated with the molecular patterns, etc [18, 19].

Regarding the size of cellular components, particles smaller than 10 μm are recognized by phagocytic cells in cellular endocytosis mechanism specifically pinocytosis, promoting antigen recognition and presenting to the APCs [14, 20] which consequently elicits a higher serum IgG response [4, 20, 21].

It should be mentioned that the natural polymeric NPs including chitosan [22, 23], gelatin [24], hyaluronic acid [25], dextran [26], pullulan [27, 28], inulin [29, 30] and sodium alginate [31] can induce cytokines and antibody responses and can also be employed as drug and antigen delivery system [18, 32]. Among natural polymeric NPs, alginate salt has growingly attracted attention for drug and antigen delivery system because of high stability in gastrointestinal tract, good moisture absorption, possible surface modification, easy fabrication, biocompatibility, biodegradability, high oxygen permeability, the lack of immunogenicity and toxicity, high loading capacity (LC) for antigen, gelation obstructive, moco‐adhesion capability, adjuvanticity, adsorption of metal ions, solubility in water and can be used to form hydrogels under cytocompatible conditions [33, 34, 35]. Alginates (ALG) are anionic polysaccharides extracted from brown algae cell walls including *Macrocystis pyrifera*, *Laminaria hyperborea*, *Ascophyllum nodosum* [36, 37] as well as several bacterial strains such as *Azotobacter* and *Pseudomonas* [38]. ALG is linear biopolymers consisting of 1,4‐linked β‐D‐mannuronic acid (M) and 1,4‐linked α‐L‐guluronic acid (G) residues arranged in homogenous (poly‐G, poly‐M) or heterogenous (MG) block‐like patterns [39]. Additionally, in M‐blocks, mannuronic moieties can be found in the ^4^C_1_ conformation, whereas guluronic residues in G‐blocks are in the ^1^C_4_ conformation based upon which chain hardness is reduced by changing MG sequences [40, 41, 42]. Concerning the composition of alginate and relative molecular mass, alginates can be classified into low viscosity, moderate viscosity and high viscosity [33]. Alginate NPs can be generated by ionic gelation method via calcium ions as well as other divalent cations. This method contains many advantages such as simple preparation process without using the organic solvent and low cost [43, 44, 45, 46]. This method forms calcium alginate complexes and interactions between the calcium ions and the glucuronic sequences of alginate polymer which leads or redounds to the egg‐box structure formation [47]. In this method, polymers are led to create hydrogels and generate a network of cross‐linked polymer chains under different diameters that can entrap different amounts of drugs and biomolecules. Moreover, the properties of the obtained gels are related to the physical condition, alginate composition in particular on the length of the G‐blocks, alginate polymer concentration and cross‐linking agent [41, 48, 49].

Based on the above‐mentioned description, this study was designed to survey the applicability of the alginate NPs as antigen delivery system loaded by diphtheria. To do so, the LC and efficiency of alginate NPs as antigen delivery system loaded by diphtheria toxoid (DT) were explored. Finally, the effect of various concentrations of sodium alginate and calcium chloride were surveyed in addition to some physical conditions such as homogenization time and rate on the NPs physicochemical characteristics.

### 1.1 Contributions

In short, the major contributions of this project can be summarised as follows:

✓ *Preparation and optimization of the alginate NPs as antigen delivery system loaded by DT*.
✓ *Investigation of the various concentrations effect of sodium alginate and calcium chloride on NPs formation*.

For this purpose, this project attempted to answer two main questions arisen as follows:

1. Does the concentration of alginate solution and CaCl_2_ cross‐linker agent have an efficient effect on the particle size formation?
2. Does the DT concentration increment may increase the Loading efficiency (LE) and LC?

This paper is organised as Section 1 for Introduction, Section 2 provides the Material and Method employed such as materials, characterisation of DT, preparation and characterisation of Alg‐NPs, loading DT in Alg‐NPs, LE and capacity, in vitro DT release and Fourier Transform Infrared (FTIR) spectroscopy. Section 3 describes the results obtained. Finally, the discussion, and the conclusions and suggestions are described in Sections 4 and 5, respectively.

## 2 MATERIALS AND METHODS

Low molecular weight sodium alginate (medium viscosity‐3500 cps) was purchased from Sigma‐Aldrich, USA. Also, calcium chloride dihydrate, Coomassie Brilliant blue G‐250, methanol 95% and phosphoric acid 85% were obtained from Merck, Germany. In addition, DT (2000 Lf/ml) was prepared from Human Bacterial Vaccine Department of Razi Vaccine and Serum Research Institute. The aqueous solutions were prepared by water for injection. It should be mentioned that all materials used were at analytical grade.

### 2.1 Characterisation of diphtheria toxoid

Before loading the DT on Alg‐NPs, the properties of toxoid such as concentration, antigenic titre and molecular weight were estimated. Afterwards, the toxoid titre and protein concentration of DT were determined by Ramon flocculation test [50] and Lowry protein assay [51]. Furthermore, the DT molecular weight was assessed by the Sodium Dodecyl Sulfate‐Polyacrylamide Gel Electrophoresis (SDS‐PAGE) method [52]. Four micrograms of DT along with the standard protein markers were briefly loaded onto 4%–12% Tris‐Glycine gels and run at 1300 mA for 5 h at room temperature and then stained with 0.1% Coomassie brilliant blue in 10% acetic acid using a solution of methanol: water at 1:1 ratio.

### 2.2 Preparation of Alg‐NPs

The preparation of Alg‐NPs was performed by ionic gelation method. Then, different concentrations of the CaCl_2_ solution (0.1%, 0.2%, 0.3% w/v) were added to various concentrations of sodium alginate solution (0.1%, 0.2%, 0.3% w/v) drop‐wisely under different homogenization rates (800, 1000, 1500 and 2000 rpm) as well as time (15, 30, 45 and 60 min) at room temperature. Next, Alg‐NPs suspensions were isolated by centrifugation (Eppendorf 5417R, Germany) at 10,000 rpm for 20 min at 4°C.

### 2.3 Alg‐NPs characterisation

The NPs were prepared and characterised for their morphology and surface appearance by SEM (LEO Electron Microscopy/Leo 440i). In addition, the NPs zeta potential and size distribution were evaluated by zeta‐sizer (Malvern Instruments) using dynamic light scattering (DLS) technique. It is worthwhile mentioning that the zeta potential (or ζ potential) denotes the electrical charge at the surface of the hydrodynamic shear surrounding the colloidal particles. Zeta potential determination is a significant characterisation technique of nano‐crystals to estimate the surface charge, which can be employed for understanding the physical stability of nano‐suspensions.

### 2.4 Diphtheria toxoid loading on Alg‐NPs

The calcium chloride aqueous solution (0.1% w/v) was added drop wise to the sodium alginate solution (0.2% w/v) containing different DT concentrations (0.5, 1, 1.5, 2, 2.5, 3 mg/ml) under 2000 rpm homogenization rate. Then, the Alg‐NPs suspension was homogenized for 30 min at room temperature.

### 2.5 Loading efficiency and loading capacity

Loading efficiency and LC were measured to assess the Alg‐NPs ability to DT entrapment in different DT concentrations, indirectly, by free DT determination in the supernatant. For this purpose, NPs suspension was centrifuged at 10,000 rpm for 20 min and then the amount of DT in the supernatant was estimated by Bradford protein assay [53]. In this approach, DT concentration was determined using a calibration curve, where the LE and LC values were calculated using the following equations [54]:

(1) $$\text{LE}\;\left(\text{\%}\right)=\frac{\text{Total}\;\text{amount}\;\text{of}\;\text{protein}-\;\text{free}\;\text{protein}}{\text{Total}\;\text{amount}\;\text{of}\;\text{protein}}\times 100$$

(2) $$\text{LC}\;\left(\text{\%}\right)=\frac{\text{Total}\;\text{amount}\;\text{of}\;\text{protein}-\;\text{free}\;\text{protein}}{\text{Dried}\;\text{weight}\;\text{nanoparticles}}\times 100$$

### 2.6 In vitro release of diphtheria toxoid

In vitro behaviour release of the DT from Alg‐NPs was determined in phosphate buffer saline (PBS) at 37°C. To do so, 3 mg of DT entrapped in NPs were equally divided in several test tubes. Afterwards, PBS (0.01 M, pH 7.4) was added to each test tube. Then, the tubes were incubated in the shaker‐incubator (Jaltajhiz, JSH20LURS, Iran) (100 cycles/min) under the temperature adjusted to 37°C. At the scheduled time intervals, samples were taken and then centrifuged at 10,000 rpm for 20 min at 4°C. Next, the amount of the DT in the supernatant was determined by Bradford assay.

### 2.7 Fourier Transform Infrared spectroscopy

In order to analyse the interactions between polymer, CaCl_2_ and DT in blank Alg‐NPs and DT‐loaded NPs, the samples were evaluated using FTIR (ALPHA II, BRUKER) at room temperature [55].

## 3 RESULTS

### 3.1 Diphtheria toxoid characteristics

**FIGURE 1:**
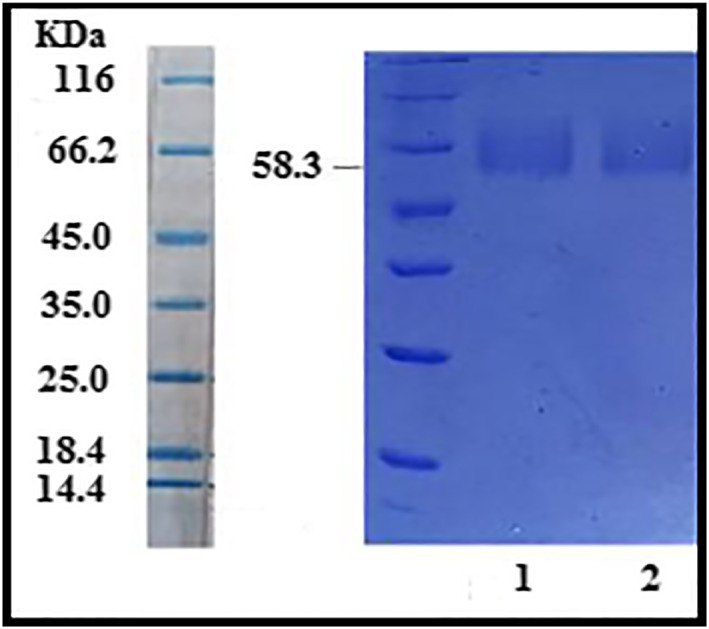
Determination of molecular weight of diphtheria toxoid by SDS‐PAGE (lane 1 and 2)

The total DT concentration and antigenic titre were obtained at 9.141 mg/ml and 2000 Lf/ml, using Bradford and Ramon assays, respectively. In addition, the molecular weight of DT was 58.3 KDa based on the SDS‐PAGE pattern, (as depicted in Figure 1).

### 3.2 Effects of different factors on NPs preparation

Several sets under the alginate concentration increment and CaCl_2_ volumes were prepared to investigate the highest concentration limit of sodium alginate in the gel‐forming in the presence of calcium ions. Table 1 shows the final composition of each set and their macroscopic evaluations. Table 2 indicates that 0.2% w/v of sodium alginate and 1.8 ml of CaCl_2_ (0.1% w/v) were the maximum limit to obtain a suitable homogeneous gel. According to Tables 3 and 4, 0.2% w/v of sodium alginate with 0.1% w/v of CaCl_2_ were achieved as the optimized condition for appropriate Alg‐NPs preparation at magnetically homogenization rate of 2000 rpm for 30 min at room temperature.

**TABLE 1:** The effect of sodium alginate and calcium chloride concentrations on the formation of nanoparticles

Calcium chloride %w/v			
Sodium alginate %w/v	0.1	0.2	0.3
0.1	CS	CS	μG
0.2	HG	μG	μG
0.3	μG	TG	Gel with lumps

Abbreviations: μG, microgel; CS, clear solution; HG, homogeneous gel; TG, thick gel.

**TABLE 2:** Gelation of sodium alginate with CaCl_2_: macroscopic and microscopic evaluations and different total solid contents in different sodium alginate concentrations

No.	Cross linker 0.1%w/v	Concentration of sodium alginate %w/v (2 ml)	Total solid content (mg/ml)	Macroscopic evaluation
1	1.4 ml	0.1	2.1	Clear solution
2	1.8 ml	0.2	4.4	Homogeneous gel
3	2 ml	0.3	6	Microgel

**TABLE 3:** Effect of the homogenization time and rate on the formation of nanoparticles

Homogenisation time (min)	15	Microgel
	30	Homogeneous gel
	45	Homogeneous gel
	60	Homogeneous gel
Homogenisation speed (rpm)	800	Thick gel
	1000	Microgel
	1500	Microgel
	2000	Homogeneous gel

**TABLE 4:** Optimised conditions to produce desirable blank Alg‐NPs

Alginate concentration (%w/v)	CaCl_2_ concentration (%w/v)	Homogenisation time (min)	Homogenisation rate (rpm)	Total solid content (mg)	Macroscopic evaluation
0.2	0.1	30	2000	4.4	Homogeneous gel

### 3.3 NPs characterisation

**FIGURE 2:**
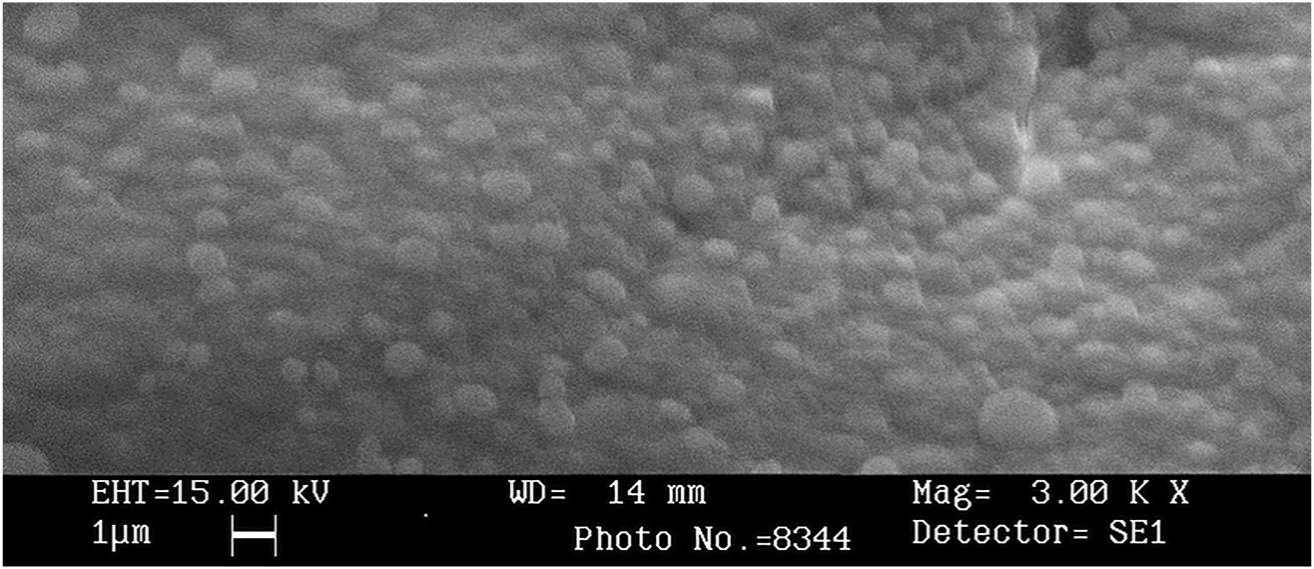
SEM image of nanoparticles

**FIGURE 3:**
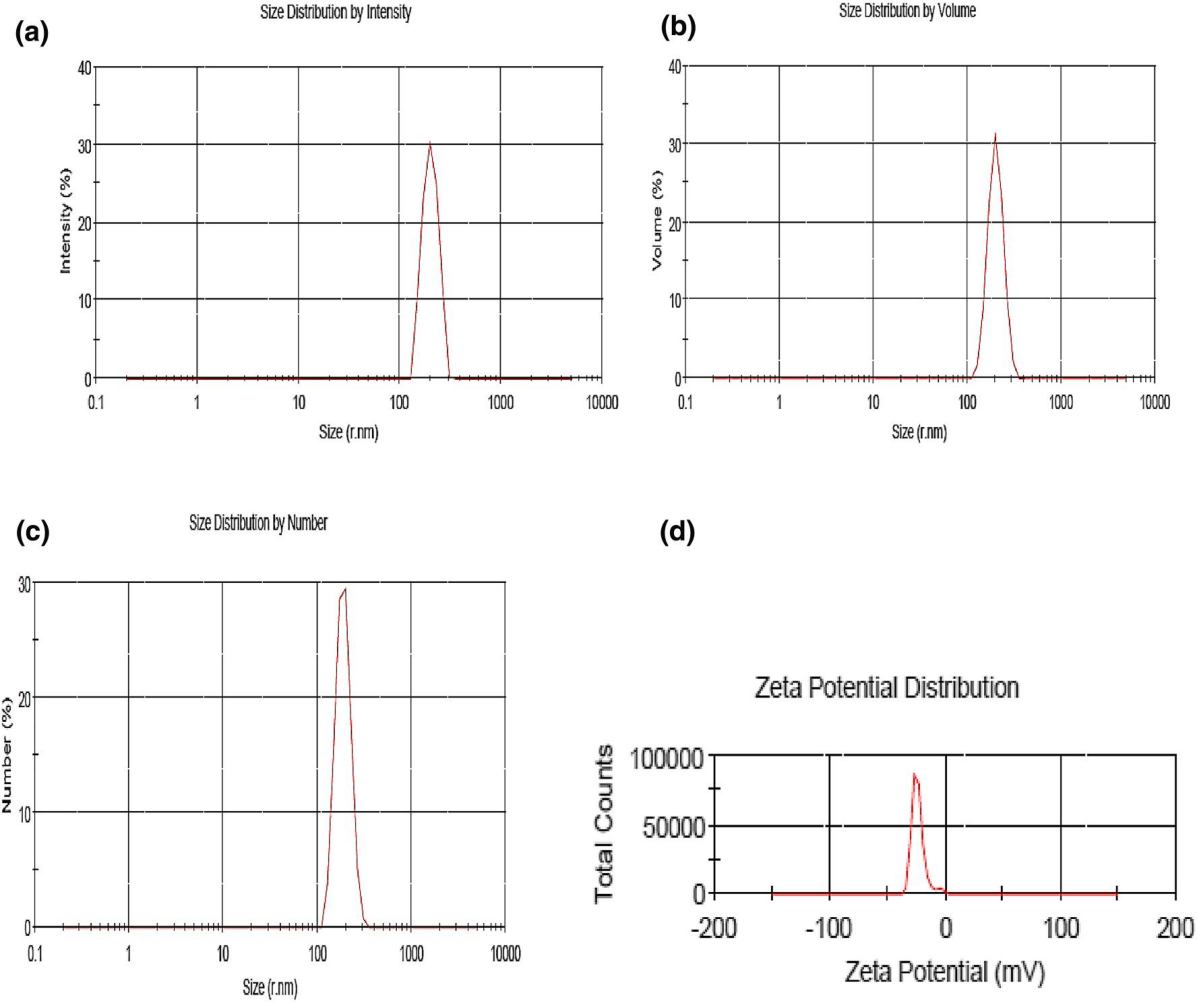
Size distribution records of blank Alg nanoparticles by (a) intensity, (b) volume, (c) number and (d) zeta potential distribution

**FIGURE 4:**
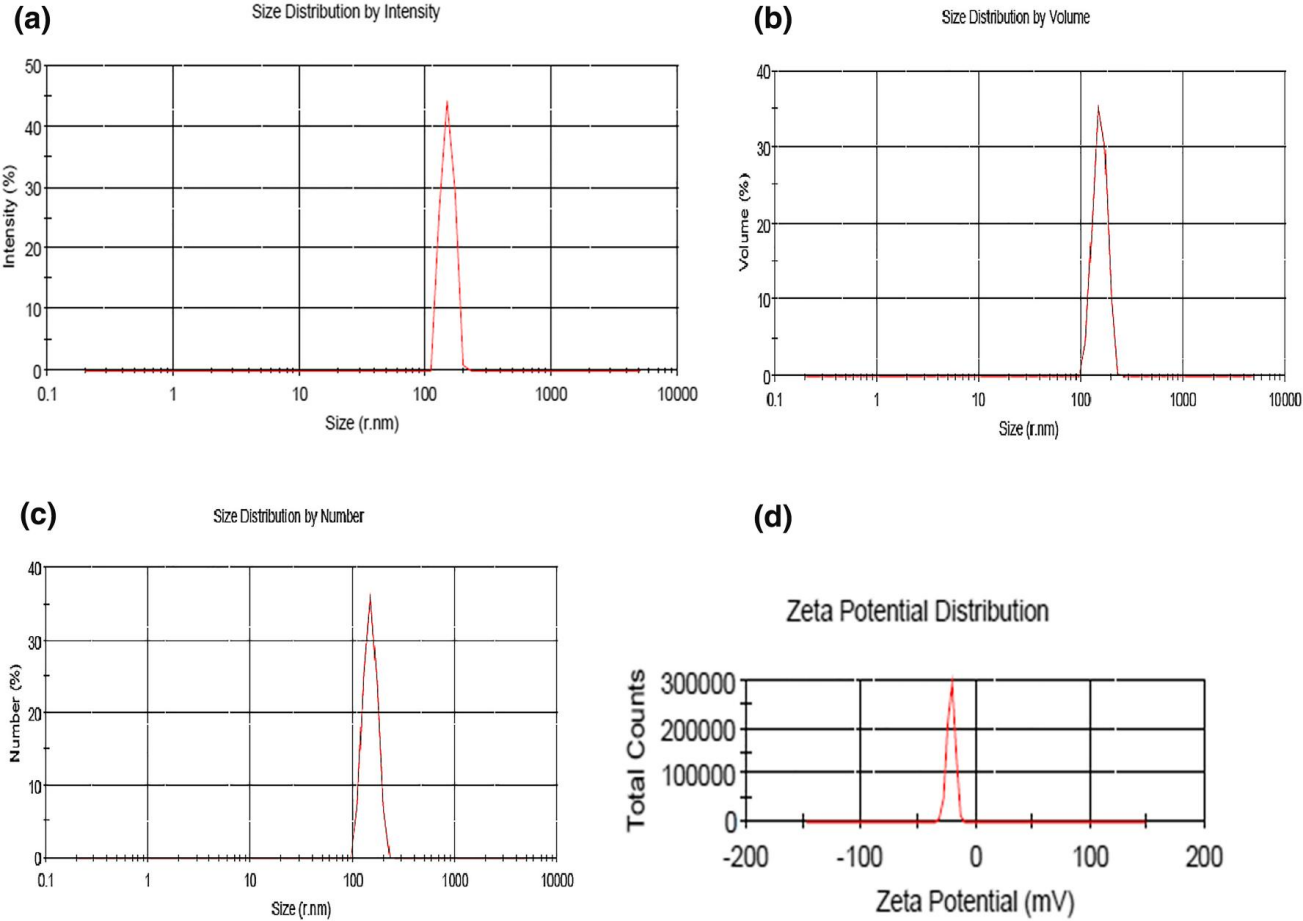
Size distribution record of diphtheria toxoid loaded Alg nanoparticles by (a) intensity, (b) volume, (c) number and (d) zeta potential distribution

As illustrated in Figure 2, the morphological characteristics of NPs were surveyed using SEM. The obtained SEM image indicated that NPs were smooth and spherical in desirable distribution. Meanwhile, results of the DLS showed that the optimized blank alginate NPs had the average size of 400.7 nm with 0.584 polydispersity index (PDI). The zeta potential of alginate NPs was also calculated as −23.7 mv (see Table 5 and Figure 3 for more details). The NPs average size was estimated 276.6 nm where the zeta potential varied to −21.2 mV after loading with DT toxoid. These NPs showed PDI of 0.493 (Table 5 and Figure 4).

**TABLE 5:** Particle size, zeta potential and PDI of the blank and DT loaded NPs

Formulations	Z‐ average mean diameter (nm)	Intensity mean diameter (nm)	Volume mean diameter (nm)	Number mean diameter (nm)	PDI	Zeta potential (mV)
Optimised blank Alg‐NPs	400.7	202.5	202.9	189.4	0.584	−23.7
DT loaded nanoparticles	276.6	149.8	154.7	149.1	0.493	−21.2

Abbreviations: DT, diphtheria toxoid; NPs, nanoparticles; PDI, polydispersity index.

### 3.4 Loading efficiency and loading capacity

**FIGURE 5:**
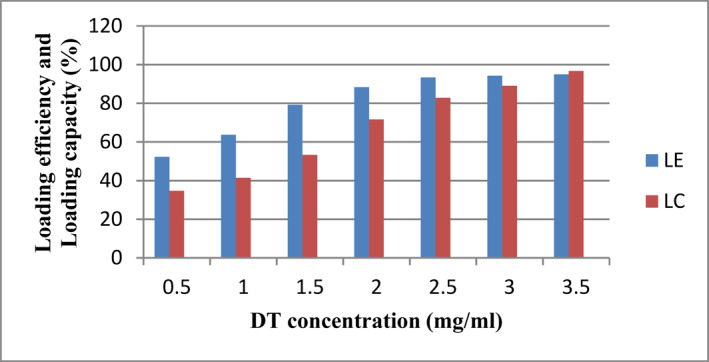
Effect of different diphtheria toxoid (DT) concentrations on the loading efficiency (LE) and loading capacity (LC) of Alg‐NPs

Different concentrations of DT were loaded in Alg‐NPs to explore the effect of antigen amount on LE and LC. As illustrated in Figure 5, by increasing the DT concentration, the LE and LC increased as well. The obtained results demonstrated that the amount of LE and LC increased significantly using 3 mg/ml DT concentration.

### 3.5 In vitro DT release

**FIGURE 6:**
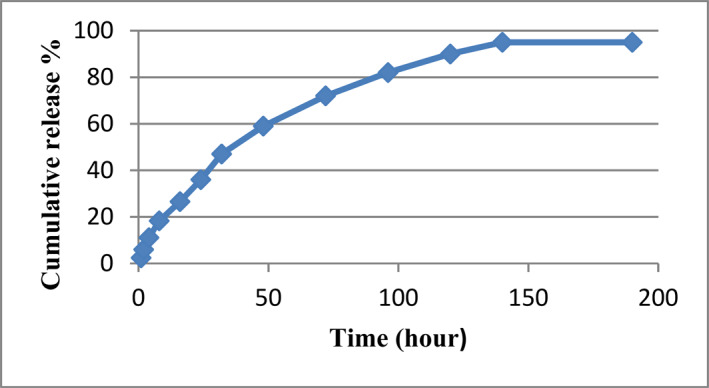
*In vitro* release profile of diphtheria toxoid from alginate nanoparticles

Release of the DT loaded Alg‐NPs was carried out using the phosphate buffer (PBS, pH 7.4) at 37°C for 190 h. According to Figure 6, the release behaviour of DT loaded Alg‐NPs was slow and continuous on the first 32 h, which had a steep slope and carried on in a constant slope until 140 h. Considering that, 95% of DT was approximately released in 140 h.

### 3.6 Fourier transform infra‐red (FTIR) measurements

**FIGURE 7:**
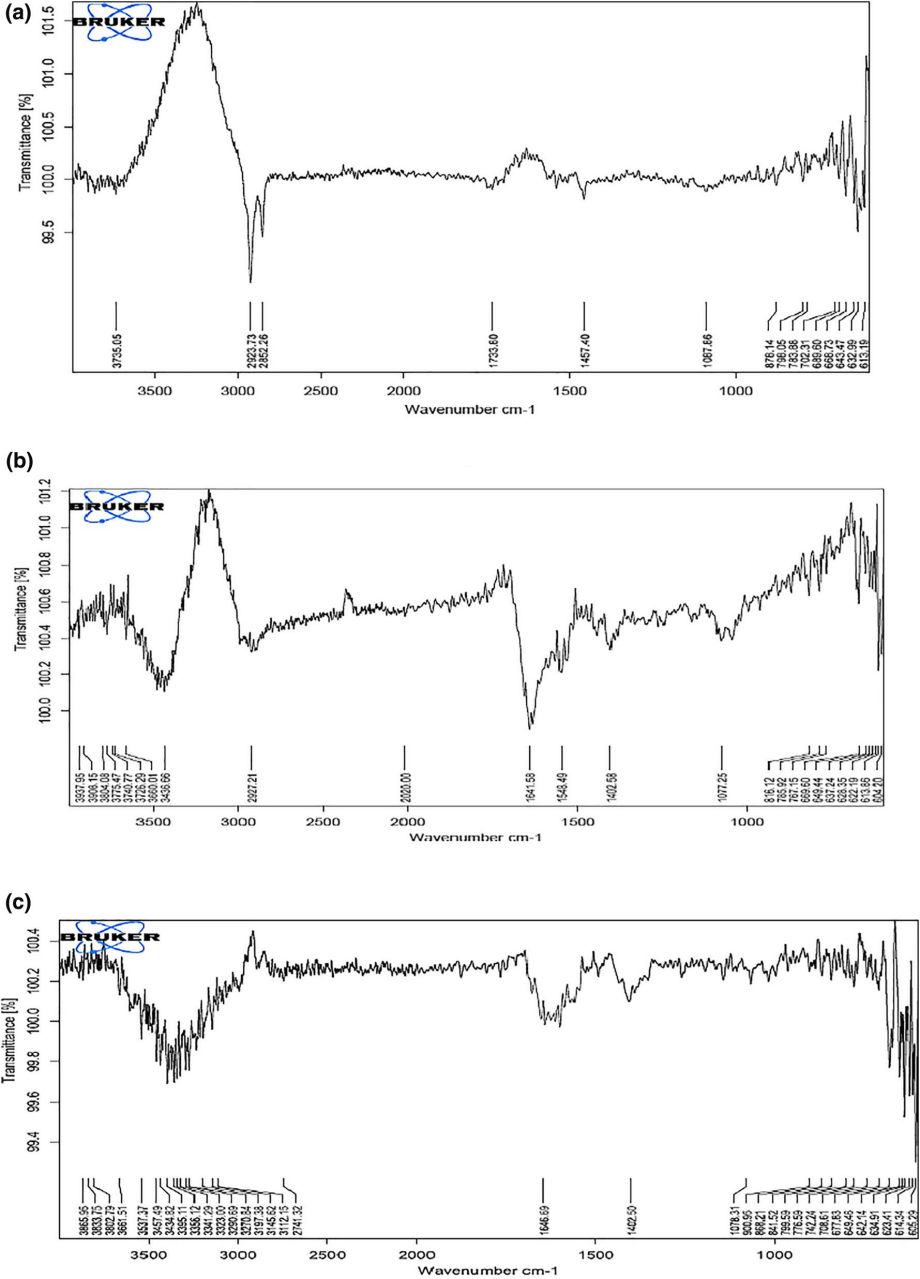
FTIR spectra of alginate and diphtheria toxoid (DT) loaded nanoparticles (NPs): (a) blank NPs (b) DT and (c) DT‐loaded alginate NPs (sodium alginate concentration 0.2% w/v, CaCl_2_ 0.1% w/v and DT 3 mg/ml)

The free and loaded NPs spectra investigation showed some changes in the amino and carboxyl groups and amide peaks, indicating an ionic interaction between the carbonyl group of alginate and the amino group of DT. The obtained FTIR results confirmed the encapsulation of DT in NPs. The FTIR spectra of alginate and DT loaded NPs are demonstrated in Figure 7.

## 4 DISCUSSION

Biocompatible NPs have received a lot of attention for antigen or drug delivery system owing to their safety and protecting antigens or drugs from the physiological and environmental changes. Alginate is a biocompatible polymer which widely used for antigen or drug delivery, protein or enzyme carrier, wound healing, tissue engineering and as matrices to immobilise cells and importantly is FDA‐approved [39, 56]. In the current study, Alg‐NPs were made up by ionic gelation method. Previously conducted studies have addressed that the concentrations of alginate solution as well as CaCl_2_ cross‐linker agent had an efficient effect on the particle size formation [57, 58, 59, 60, 61]. To understand how the effect of different concentrations of CaCl_2_ and sodium alginate affects the formation of Alg‐NPs, different concentrations of CaCl_2_ (0.1%, 0.2% and 0.3% w/v) and sodium alginate (0.1%, 0.2% and 0.3% w/v) were employed for Alg‐NPs preparation in primary screening. The optimized formulation was chosen based on the suitable morphology in terms of some items such as particle size and poly dispersity index, high protein loading and sustained release. In the current research, 0.2% w/v of sodium alginate and 0.1% w/v of CaCl_2_ were appropriate concentrations for preparing the optimized Alg‐NPs in terms of above‐mentioned criteria.

As listed in Table 1, an increment in sodium alginate concentration of more than 0.2% w/v would lead to the development of viscosity and insufficiency of enough shear stress, which facilitates the macroscopic gel aggregates formation as well as Alg‐NPs particle size increment. On the other hand, an important factor in forming of NPs is the orientation of functional groups of alginate chains such as carboxylate groups to make a complex structure associated with calcium ions. Accordingly, in conjunction with increasing in the alginate concentration, more functional groups can be obtained, such that neighbouring calcium ions can be collected, leading more layers of alginate chains link the calcium ions. Subsequently, the more alginate concentration is led to the more increase in Alg‐NPs size. In contrast, increasing the CaCl_2_ concentration would result in an imbalance between Ca^2+^ and alginate binding sites and, lead to lower numbers of the polymer chains involved in higher contents of calcium ions. Therefore, the cross linking between the glucoronic acid residues and calcium ions was developed that formed the microscopic gel aggregates as well as the Alg‐NPs smaller size in conjunction with increasing of CaCl_2_ concentration. Here, it is worthwhile mentioning that the results of the current research were in accordance with previous conducted studies, which had described an increasing in sodium alginate and CaCl_2_ concentrations would lead to increase and decrease in Alg‐NPs size formation, respectively [32, 57, 58, 59, 60, 61].

Effects of certain processing parameters such as homogenization time and rate on Alg‐NPs size were evaluated. Our results showed that an increase in homogenization time from 15 to 60 min would lead to a decrease in the mean particle size. Other similar studies had correspondingly shown that increasing in the homogenization time had ultimately been led to a decrease in the particle size [32, 57, 58]. On the other hand, by increasing the homogenization rate from 800 to 2000 rpm, the Alg‐NPs size was decreased. Meanwhile, similar results have been reported in previous studies [32, 57, 62, 63]. So, these results illustrated that homogenization rate of 2000 rpm at 30 min might be an optimized condition for Alg‐NPs preparation (Table 3).

The results determined by DLS demonstrated that size distribution of NPs before and after loading DT was achieved in a narrow range and only one peak was observed (Figures 3 and 4). Nanoparticles in the range of 200–500 nm are at appropriate size for antigen or drug delivery [17]. The NPs adherence to the cell membranes and immune cells uptake depends on the surface charge. Accordingly, conversion of the particle surface charge could control binding to the cells [64]. Electrophoretic mobility of particles and the total electric charge on the particles surface are determined by zeta potential technique. Zeta potential is in particular important for NPs in suspension as a key role in interaction between the drug or antigen and NPs, and as the suspension stability indicative and particle surface morphology [65, 66, 67, 68]. The zeta potential of Alg‐NPs was obtained −23.7 mV and then changed to −21.2 mV (Figure 3d) after DT loading (Figure 4d). This negative zeta potential value for Alg‐NPs and DT‐loaded NPs demonstrates the high surface charge of prepared NPs. These surface charges lead to high suspension stability for Alg‐NPs before and after encapsulation with DT through strong repellent interaction between NPs in the dispersion. On the other hand, it should be noted that a reduction in the zeta potential value was due to the encapsulation of DT because of interaction between the negative carboxyl (COO^−^) groups on Alg‐NPs, which neutralised by positive (NH^2+^) groups on DT, leading to DT‐Alg‐NPs formation. As a result, this alteration indicated that the DT encapsulation was successfully performed. Afterwards, different concentrations of DT were loaded in NPs to investigate the ability of Alg‐NPs for encapsulating antigens as well as the effect of the antigen amount on LE and LC. Thus, by increasing the DT concentration, the LE and LC were increased as well. Furthermore, created NPs in the current study with high concentration of DT showed significant LE (>80%) and LC (>90%) (Figure 5). Finally, a suitable concentration for preparing optimum Alg‐NPs was achieved at 3 mg/ml DT concentration. It should be noted that the higher DT encapsulation efficiency of Alg‐NPs might be due to the interaction of DT with hydroxyl groups of alginate chains via the formation of hydrogen and electrostatic bounds on the unsaturated sites in alginate chains. As the DT and polymer had the same solvent (water), the antigen cannot diffuse from the polymer [69]. Accordingly, by increasing the DT concentration, free binding sites in alginate chains are saturated with DT and leave not adequate space to entrap the antigen. Therefore, LE did not increase in parallel to DT concentration in higher than 3 mg/ml. The similar observations have previously been reported in other studies [49, 70, 71].

The main mechanism for antigen release from Alg‐NPs is carried out by the diffusional processes through pores comforted by the polymeric network degradation [72, 73]. In this study, *In vitro* release of the DT from Alg‐NPs was a biphasic linear profile with long release time (as depicted in Figure 6). The first phase was very fast that was followed by a slow release. The initial fast release could be interpreted as due to the free DT adsorbed on the Alg‐NPs surfaces. Furthermore, the sustained release was related to the cleavage of the chemical and electrostatic bonds between DT and sodium alginate particles [72, 74]. The DT‐loaded NPs prepared in the current study owing to the good sustained release without burst release can be used as candidate for antigen delivery.

The FTIR spectra of the sodium alginate NPs, DT and DT‐loaded NPs were compared in Figure 7. According to the FTIR spectra, a low difference can be observed in the width and frequency of the peaks, in which the wide peak obtained in 3400 cm^−1^ is corresponding to O‐H stretching and intermolecular hydrogen bonding. The wave of 1402–1640 cm^−1^ is matching with the C=O stretching (amide). Even though, stretching vibration of aliphatic C‐H was observed at 2850–2920 cm^−1^. The carboxyl peaks were seen adjacent to 1646 cm^−1^ (symmetric –COO− stretching vibration) and 1415 cm^−1^ (asymmetric COO– stretching vibration), which were expanded after interaction with DT. Comparison of the spectra for frees and loaded NPs showed an ionic interaction between the carboxyl group of alginate and the amino group of DT. As a consequence, the FTIR results confirmed the successful interaction of Alg‐NPs and DT.

## 5 CONCLUSION

In the current study, the alginate NPs were prepared and optimized. Moreover, the influence of various concentrations of sodium alginate and calcium chloride were investigated in addition to some physical conditions such as homogenization time and rate on the NPs physicochemical characteristics. This study described that alginate could be employed as a static environment for controlling drug release at an advantageous rate. For this purpose, the alginate NPs were synthesised by the addition of calcium chloride solution to the diluted solutions of sodium alginate through the controlled ionic gelation method. The obtained results showed that the features of NPs such as morphology and particle size were strongly dependent on the calcium chloride and sodium alginate concentrations as well as physicochemical conditions in the NPs formation process. Moreover, the suitable concentrations of sodium alginate and calcium ions would lead to obtaining the desirable NPs formation via desirable LE and LC (over 80%) and sustained *in vitro* release profile. Also, the effect of other processing parameters such as homogenization time and homogenization rate was investigated on the NPs size. According to the obtained results, as the homogenization time increased from 15 to 60 min, the particle size inversely decreased.

For further research, it is suggested that the DT‐loaded on sodium alginate NPs could be utilised as a proper system for DT antigen delivery. Moreover, it can be employed to produce monovalent diphtheria NP vaccine or in combination with other bacterial NP vaccines for prevention as well as in cancer immunotherapy conjugated with anti‐CDs. Furthermore, the proposed sodium alginate NPs along with the formulation of cross‐reacting recombinant diphtheria antigens such as CRM_197_, could be used as a new vaccine platform.

## CONFLICT OF INTEREST

The authors declare that they have no conflict of interest(s).

## CREDIT CONTRIBUTION STATEMENT

Not applicable.

## Data Availability

Almost all documents related to the current research on preparation and characterization of the NPs and data analyzed during this study are entirely included.
